# RELATIONSHIPS OF SOCIAL AND COGNITIVE ACTIVITY TO WELL-BEING AND COGNITIVE FUNCTIONING OVER TIME

**DOI:** 10.1093/geroni/igad104.0322

**Published:** 2023-12-21

**Authors:** Hsiao-Wen Liao, Li Chu, Laura Carstensen

**Affiliations:** Georgia Institute of Technology, Atlanta, Georgia, United States; Stanford University, Stanford, California, United States; Stanford, Stanford, California, United States

## Abstract

Research on engaged lifestyles points to promising ways to age well. Findings are mixed, however, when different types of social and cognitive activity engagement are examined. The present study, juxtaposing core tenets of socioemotional selectivity theory and the engagement hypothesis, tested the relative importance of social engagement with close partners and engaging in cognitively stimulating activities for older adults’ psychological well-being and cognitive performance over time. Of particular interest was the comparison between individuals with high levels of interaction with close social partners yet low levels of cognitive activity engagement and those with low levels of social engagement but high levels of cognitive activity engagement. The sample (N = 1607) was drawn from participants in the Health and Retirement Study who participated in the 2008, 2012, 2016, and 2020 modules. Engagement profiles were created based on the frequency of contact with close social partners outside the household (i.e., children, family members, and friends) and engagement in cognitively stimulating activities (i.e., reading, writing, using computers, cooking, playing word games, and doing hobbies) at baseline. Results of growth curve modeling, controlling for demographic background and baseline performance, showed that, as predicted, older adults with high levels of social interactions and low levels of cognitive activity engagement had superior life satisfaction but poorer performance on delayed recall than their counterparts who had the opposite engagement profiles. We draw on the life-span principle that development involves gains and losses in interpreting the findings.

